# Complementary Log Regression for Sufficient-Cause Modeling of Epidemiologic Data

**DOI:** 10.1038/srep39023

**Published:** 2016-12-13

**Authors:** Jui-Hsiang Lin, Wen-Chung Lee

**Affiliations:** 1 Research Center for Genes, Environment and Human Health and Institute of Epidemiology and Preventive Medicine, College of Public Health, National Taiwan University, Taipei, Taiwan

## Abstract

The logistic regression model is the workhorse of epidemiological data analysis. The model helps to clarify the relationship between multiple exposures and a binary outcome. Logistic regression analysis is readily implemented using existing statistical software, and this has contributed to it becoming a routine procedure for epidemiologists. In this paper, the authors focus on a causal model which has recently received much attention from the epidemiologic community, namely, the sufficient-component cause model (causal-pie model). The authors show that the sufficient-component cause model is associated with a particular ‘link’ function: the complementary log link. In a complementary log regression, the exponentiated coefficient of a main-effect term corresponds to an adjusted ‘peril ratio’, and the coefficient of a cross-product term can be used directly to test for causal mechanistic interaction (sufficient-cause interaction). The authors provide detailed instructions on how to perform a complementary log regression using existing statistical software and use three datasets to illustrate the methodology. Complementary log regression is the model of choice for sufficient-cause analysis of binary outcomes. Its implementation is as easy as conventional logistic regression.

The logistic regression model is the workhorse of epidemiological data analysis^1^. The model helps to clarify the relationship between multiple exposures and a binary outcome. Researchers can easily adjust for confounding factors and assess interactions by entering appropriate covariates into a logistic regression model. An (exponentiated) regression coefficient of a main-effect term in the model is an adjusted odds ratio, and a test of the regression coefficient of a cross-product term is a test for multiplicative interaction. Logistic regression analysis is readily implemented using existing statistical software, and this has contributed to it becoming a routine procedure for epidemiologists.

Logistic regression is a generalized linear model with a ‘logit’ link function^2^. (A link function specifies how the exposure variables are related to the mean response.) Statistics textbooks often describe two other link functions for a binary outcome, the ‘probit’ and the ‘complementary log-log’ links, though these two link functions are less often used in epidemiology.

In this paper, we focus on a causal model which has recently received much attention from the epidemiologic community, namely, the sufficient-component cause model (causal-pie model)^1^,^3^,^4^,^5^,^6^,^7^,^8^,^9^,^10^,^11^. The model is mechanism-based, aiming at elucidating the possible mechanisms through which multiple exposures interact in causing an outcome. We will show that the sufficient-component cause model can be associated with yet another link function, the ‘complementary log’ link, and that in a complementary log regression, the exponentiated coefficient of a main-effect term corresponds to an adjusted ‘peril ratio’ (a recently introduced alternative measure for exposure effect^9^,^10^), and the coefficient of a cross-product term can be used directly to test for causal mechanistic interaction (sufficient-cause interaction). While a number of previous researchers have considered such a link function, they were unaware of its correspondence to the sufficient-component cause model^12^,^13^,^14^,^15^,^16^,^17^,^18^. To promote complementary log regression for epidemiologic data analysis, we provide detailed instructions on how to perform such a regression using existing statistical software and use three datasets to illustrate the methodology.

## Methods

We are interested in the relationship of two binary exposures (*X* and *Z*) and a binary outcome. We assume that in a cohort study of a population in a certain time interval, (0, *T*), the exposure status is time-invariant, and the follow-up is fully complete, that is, without loss to follow-up and competing death. We assume that there is no confounding, selection bias, or measurement error in the study. Therefore, the association between the two exposures and the disease should reflect the genuine causal effects of the exposures on the disease.

**Figure 1:**
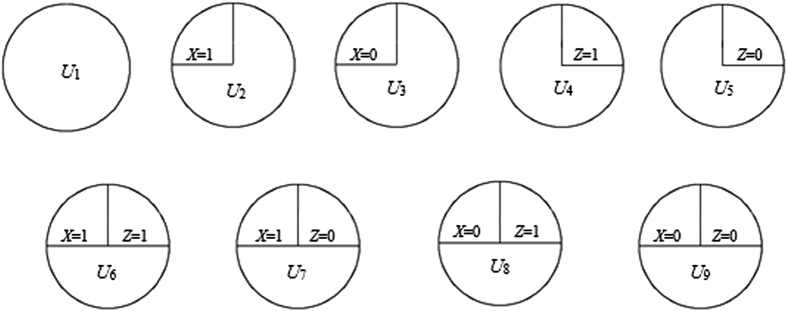
All 9 classes of sufficient cause for two binary exposures.

For two binary exposures, there is a total of nine classes of sufficient causes, including one ‘all-unknown’ class (*U*_1_), two *X*-only classes (*U*_2_, *U*_3_), two *Z*-only classes (*U*_4_, *U*_5_), and four interaction classes (*U*_6_-*U*_9_) (see Fig. 1)^8^,^9^. For people in the population with an exposure profile of *X* = *x* and *Z* = *z* for each and every *x, z* ∈ {0, 1}, let Risk_*x, z*_ denote the cumulative disease risk (probability) in (0, *T*), and Peril_*x, z*_ = (1 − Risk_*x, z*_)^−1^, the disease peril in (0, *T*). [‘Peril’ is an alternative metric for risk. It is the inverse of a survival (risk complement) and ranges from 1 (no peril) to infinity (maximum peril). See ref. 9 for more details].

Furthermore, let 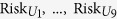 denote the cumulative completion risks of the above mentioned nine classes of sufficient causes in (0, *T*), and 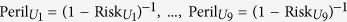, the corresponding completion perils, respectively. Under the no redundancy assumption^8^,^19^, Lee^9^ showed that the log disease peril for a specific exposure profile is the sum of log completion perils for four classes of sufficient causes, that is,


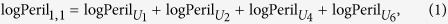



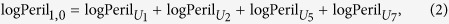



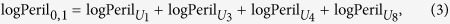


and


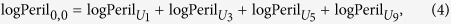


respectively.

A log peril is, in fact, a risk in complementary log transform, that is, log Peril = −log(1 − Risk). Equations (1, 2, 3, 4) above therefore suggest a complementary log regression model for disease risks:


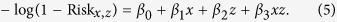


The exponentiated negative beta coefficients of this model, respectively, can be related to disease perils, as detailed below:


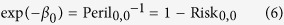


is the inverse of the peril of the reference group, which is also the survival probability of an (*X* = 0, *Z* = 0) person,


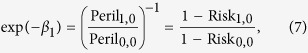


is the inverse of the peril ratio for exposure *X*, which is also the ratio of the survival probabilities between an (*X* = 1, *Z* = 0) person and an (*X* = 0, *Z* = 0) person,


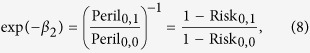


is the inverse of the peril ratio for exposure *Z*, which is also the ratio of the survival probabilities between an (*X* = 0, *Z* = 1) person and an (*X* = 0, *Z* = 0) person, and finally,


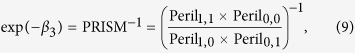


is the inverse of the ‘peril ratio index of synergy based on multiplicativity’ (PRISM) proposed by Lee^9^.

From Equations (1, 2, 3, 4), the PRISM is also related to the completion perils:


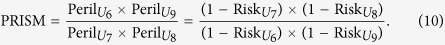


Because PRISM ≠ 1 in Equation (10) [or equivalently, PRISM^−1^ ≠ 1 in Equation (9)] forbids 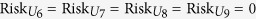, a test for the interaction term (H_0_: *β*_3_ = 0 against H_1_:* β*_3 _≠ 0) in the complementary log regression model [Model (5)] can therefore be taken directly as a test for causal mechanistic interactions (sufficient-cause interactions) between *X* and *Z*, that is, a test for the presence of at least one of the interaction classes (*U*_*6*_-*U*_*9*_). [Model (5) also permits hypothesis testing for specified interaction classes. See S1 Exhibit for details.] By comparison, to conduct the same PRISM test based on other models (logistic, probit, and complementary log-log) requires much more computational effort (S2 Exhibit) than was needed here with complementary log regression.

For the general situation of multiple multi-leveled exposures (two or more exposures, each with two or more levels), the above proposed complementary log regression model also applies. The exponentiated negative beta coefficient for an exposure level in the model is an estimate of the ratio of the survival probabilities between a person with that exposure level and one with the reference level, and a test of the beta coefficients of the *k*^th^-order interaction terms involving a particular set of a total of *k (k* ≥ 2) exposures is a test for the *k*^th^-order causal mechanistic interactions among this exposure set. As for a continuous exposure, it can be categorized into multiple levels and then enter the complementary log regression as a multi-leveled exposure. Alternatively, it can enter the regression as it is, if it is reasonable to assume that the fold change in survival probability per unit change in the exposure is everywhere the same in its possible levels.

The complementary log regression model can be readily implemented using existing statistical software. For example in the SAS statistical software package (SAS Institute, Inc., Cary, North Carolina), one can select the generalized linear model (GENMOD) procedure, and specify either the link function as *g*(*μ*) = −log(1 − *μ*) in the FWDLINK statement or the inverse link function as *μ* = 1 − exp(−*g*(*μ*)) in the INVLINK statement. S3 Exhibit shows how to use the function glm() to fit the complementary log model in the R statistical software package (R Foundation for Statistical Computing, Vienna, Austria). For both software package, one also has the option of trying different sets of initial values, if an attempt to fit the model fails to converge.

## Examples

To illustrate the methodology, we fit the complementary log regression to three datasets.

### Example 1: Causal Mechanistic Interaction between Age and BMI on Hypertension

The first example is composed of cohort data taken from Example 3 in Zou’s paper^20^. A total of 4897 participants were followed up to investigate the effects of age (coded as 1 and labeled “old” if ≥40 years and coded 0 and labeled “young” otherwise) and body mass index (BMI, coded as 1 and labeled “high” if ≥25 kg/m^2^ and coded 0 and labeled “low” otherwise) on hypertension (coded as 1 and labeled “hypertensive” if diastolic blood pressure ≥90 mmHg and coded 0 and labeled “non-hypertensive” otherwise). During the follow-up, a total of 610 subjects were diagnosed to be hypertensive.

The complementary log regression was fit to the data. Table 1 presents the regression coefficients and the 95% confidence intervals (CIs). The exponentiated negative intercept is exp(−0.0446) = 0.9564 (95% CI: 0.9470–0.9658). This means that subjects in the reference group (i.e., those who are young and have a low BMI) have a 95.6% probability of being hypertension-free during the follow-up. The exponentiated negative regression coefficient for age is exp(−0.1142) = 0.8921 (95% CI: 0.8634–0.9217). This is the inverse of the peril ratio for age, which implies that the probability of being hypertensive-free for an *old* person with low-BMI is 0.89-fold that of the hypertensive-free probability for a *young* low-BMI person. Likewise, the inverse of the peril ratio for BMI is exp(−0.0724) = 0.9302 (95% CI: 0.9108–0.9499), which implies the hypertensive-free probability for a *high*-BMI young person is 0.93-fold that of the hypertensive-free probability for a *low*-BMI young person.

The exponentiated negative coefficient for the cross-product term is exactly the inverse of PRISM index [9]: exp(−0.0866) = 0.9170(95% CI of 0.8696–0.9671). Because the test for this cross-product term is highly significant (two-sided *P* value of 0.0014), we conclude that there is a significant causal mechanistic interaction between age and BMI on hypertension. Actually, the inferences are exactly the same as in Lee’s paper^9^ where the same data was also analyzed. However, the approach taken here is simpler since all the computations can be relegated to user-friendly statistical software (such as SAS and R).

### Example 2: Effects of Age and Tolbutamide Treatment on All-Cause Mortality

The second example considers randomized, controlled trial data taken from Table 15–1 in the textbook *Modern Epidemiology*^1^. A total of 409 diabetic patients were followed up to compare all-cause mortality (coded as 1 if dead from any cause and 0 otherwise) of patients treated with tolbutamide and those given a placebo (coded as 1 if in the tolbutamide treatment group and 0 otherwise), stratified by age (coded as 1 if age ≥55 years and 0 otherwise). A total of 51 subjects died during the follow-up.

Table 2 presents the results of the complementary log regression. The exponentiated negative intercept is exp(−0.0426) = 0.9583 (95% CI: 0.9232–0.9947). This means that the reference group (a patient <55 years old) has a survival probability of 95.8% during the follow-up. In this example, age is found to be the only significant determinant of all-cause mortality for diabetic patients (two-sided *P* value of 0.0028), upon adjusting for the treatment group and the cross-product term. The inverse of the peril ratio for age is exp(−0.1660) = 0.8470 (95% CI: 0.7601–0.9446), implying that the survival rate for *old* patients in the placebo group is 0.85-fold that of the survival for *young* patient in placebo group. Note that the test for the cross-product term here produces the same insignificant result (two-sided *P* value = 0.9057) as did the ‘heterogeneity test’ in a previous paper^10^.

### Example 3: Effects of Age and Personality on Coronary Heart Disease Occurrence

The third example considers cohort data taken from Table 7–24 in the textbook *Statistical Analysis of Epidemiology Data*^21^. A total of 3154 participants were followed up to compare the occurrence of coronary heart disease (CHD, coded as 1 if diseased and 0 otherwise) in personality types A and B (coded as 1 if personality type A and 0 otherwise), stratified by age (coded as 0, 1, 2, 3, and 4 if age <40, 40–44, 45–49, 50–55, and ≥55 years, respectively). During the follow-up, a total of 257 subjects acquired CHD.

Table 3 presents the results of the complementary log regression for this example. The likelihood ratio test comparing the full model to a reduced model without the interaction term is not significant [two-sided *P* value = 0.1605, based on a test statistic of 2 × (854.3623 − 851.0780) = 6.5686 with a degree of freedom of 9 − 5 = 4]. We therefore conclude that there is no causal mechanistic interaction between personality type and age.

The exponentiated intercept is exp(−0.0398) = 0.9610. This means that participants in the reference group (those younger than 40 years and of personality type A) have a 96.1% probability of remaining CHD-free during the follow-up. The inverse of the age-adjusted peril ratio for personality (type A vs. type B) is exp(−0.0399) = 0.9609. This is interpreted to mean that an A-type’s probability of remaining CHD-free is 0.96-fold that of a type B person of the same age.

On the other hand, the personality-adjusted peril ratios for age (using ages <40 as the reference age range) are: exp(0.0039) = 1.0039 for ages 40–44, exp(−0.0196) = 0.9806 for ages 45–49, exp(−0.0486) = 0.9526 for ages 50–54, and exp(−0.362) = 0.9644 for ages ≥55, respectively. These are to be interpreted as the fold-differences in the probability of remaining CHD-free for an older person in the given age range (of a particular personality type) as compared to a person in the youngest reference age range (of the same personality type).

Lee^10^ previously analyzed the same data and reached the same conclusion of no causal mechanistic interaction between personality type and age. But the method he used requires computationally challenging matrix calculations. Using a stratified analysis method, Lee^10^ also calculated the age-adjusted peril ratio for personality type (a binary exposure variable). But a stratified analysis method for the adjusted peril ratios for a polychotomous exposure variable (e.g., the personality-adjusted peril ratios for different age groups) is currently lacking.

## Discussion

In this paper, we show that complementary log regression analysis is tantamount to sufficient-component cause modeling. Under such a regression framework, an exponentiated main-effect coefficient is an adjusted peril ratio, and a test for an interaction coefficient is itself a test for causal mechanistic interaction. This should greatly facilitate our elucidation of how complex interactions between multiple risk factors bring about an outcome. Caution should be exercised when using the model to make predictions, though, as the link function does not guarantee the predicted risk for a new subject to fall between 0 and 1, if he/she is too ‘dissimilar’ to those subjects used for building the model. (By too dissimilar, we mean that the new data point is not in the convex set constructed by the data used for building the model.) To prevent this from happening, we can modify the model for risk prediction as 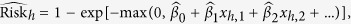 where 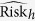 is the predicted risk, and *x*_*h*,1_, *x*_*h*,2_, … are the covariates (possibly including interaction terms) for the new subject, and 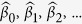 are the estimated regression coefficients of the complementary log regression. By contrast, the logistic, the probit, and the complementary log-log regressions do not suffer from this problem, because their link functions map the unit interval (for a risk) onto the real line (for a linear combination of multiple exposures).

We have focused here on a cohort population with a fixed time interval over which the exposure status is time-invariant and the follow-up is fully complete. Under this setting, the conventional ‘relative excess risk due to interaction’ (RERI) index^22^,^23^,^24^,^25^,^26^ can be used to assess causal mechanistic interactions. A RERI test (H_0_: RERI ≤ 1 against H_1_: RERI > 1) is a specific test for the (*X* = 1, *Z* = 1) interaction class^22^,^23^,^24^,^25^,^26^. However, Lee^9^ has pointed out that the RERI test uses a more stringent threshold (and hence has a lower power) to detect causal mechanistic interactions than does the PRISM test or, equivalently, a test based on the coefficient of a cross-product term in a complementary log regression (*β*_3_ in Model (5)). S4 Exhibit further shows that the complementary log regression also applies to a ‘sub-cohort study’ which randomly selects study subjects at one point in time from a source population for cross-sectional survey and subsequent follow-up.

When the outcome is rare, risk ratios can be approximated by odds ratios, which in turn can be estimated from a case-control study. Therefore, a RERI test (in terms of odds ratios) can be a valid test (or approximately so) for causal mechanistic interactions in case-control studies for rare diseases^22^,^23^,^24^,^25^,^26^. Similarly, we have that for rare diseases, log Peril = −log(1 − Risk) ≈ Risk ≈ Odds. Our Model (5) thus becomes a linear model for the odds: Odds_*x*,*z*_ = *β*_0_ + *β*_1_*x* + *β*_2_*z* + *β*_3_*xz*, or alternatively, a linear model for the odds ratios (ORs): 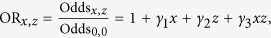 where *γ*_1_ = *β*_1_/*β*_0_, *γ*_2_ = *β*_2_/*β*_0_ and *γ*_3_ = *β*_3_/*β*_0_. Therefore, the complementary log model also applies to case-control studies for rare diseases. In this model, the main-effect coefficient, *γ*_1_ (*γ*_2_), is the excess odds ratio (OR − 1) for the *X*(*Z*) variable^27^, and the interaction coefficient, *γ*_3_, is OR_1,1_ − OR_1,0_ − OR_0,1_ + 1, which is the RERI index (in terms of the odds ratios for the interaction effects). For case-control studies of common diseases, however, neither the proposed complementary log model nor a linear risk model (for the RERI test) applies [See S5 Exhibit for details].

In summary, complementary log regression is the model of choice for sufficient-cause analysis of binary outcomes. Its implementation is as easy as conventional logistic regression. However, complementary log regression assumes a complete follow-up. Further studies are warranted to develop sufficient-cause modeling methods for censored data^28^.

## Additional Information

**How to cite this article**: Lin, J.-H. and Lee, W.-C. Complementary Log Regression for Sufficient-Cause Modeling of Epidemiologic Data. *Sci. Rep.* **6**, 39023; doi: 10.1038/srep39023 (2016).

**Publisher’s note:** Springer Nature remains neutral with regard to jurisdictional claims in published maps and institutional affiliations.

## Supplementary Material

Supplementary Information

## Figures and Tables

**Table 1:** The results of the complementary log regression for Example 1.

Variables	Regression coefficients	95% confidence interval	*P* (two-sided)
Intercept	0.0446	0.0348, 0.0545	<0.0001
Age	0.1142	0.0815, 0.1469	<0.0001
BMI	0.0724	0.0514, 0.0934	<0.0001
Age × BMI	0.0866	0.0335, 0.1397	0.0014

**Table 2:** The results of the complementary log regression for Example 2.

Variables	Regression coefficients	95% confidence interval	*P* (two-sided)
Intercept	0.0426	0.0053, 0.0799	0.0254
Age	0.1660	0.0570, 0.2743	0.0028
Treatment	0.0359	−0.0300, 0.1019	0.2859
Age × Treatment	0.0098	−0.1520, 0.1716	0.9057

**Table 3:** The results of the complementary log regression for Example 3.

Variables	Regression coefficients	95% confidence interval	*P* (two-sided)
Intercept	0.0398	0.0163, 0.0633	0.0009
Age			
<40	0.0000		
40–44	−0.0039	−0.0319, 0.0242	0.7879
45–49	0.0196	−0.0150, 0.0543	0.2664
50–54	0.0486	0.0004, 0.0967	0.0480
≥55	0.0362	−0.0187, 0.0911	0.1966
Personality Type	0.0399	−0.0022, 0.0821	0.0631
Age × Personality			
<40	0.0000		
40–44	−0.0049	−0.0557, 0.0459	0.0631
45–49	0.0364	−0.0258, 0.0986	0.8514
50–54	0.0387	−0.0410, 0.1185	0.2514
≥55	0.0898	−0.0032, 0.1828	0.3412
