# Supplementary material for: Complementary Log Regression for Sufficient-Cause Modeling of Epidemiologic Data
Source: Sci Rep. 2016 Dec 13;6:39023. doi: 10.1038/srep39023 (PMC5154187; doi:10.1038/srep39023)
Supplement: Supplementary Information [file srep39023-s1.pdf]

Title: Complementary Log Regression for Sufficient-Cause Modeling of Epidemiologic Data

Authors: Jui-Hsiang Lin, Wen-Chung Lee

Supplementary information:

S1 Exhibit. Hypothesis testing for specified interaction classes in a complementary log regression.

S2 Exhibit. Conducting the PRISM test based on the logistic, the probit, and the complementary log-log regressions.

S3 Exhibit. R code for complementary log regression.

S4 Exhibit. Applicability of the complementary log regression for a sub-cohort study which randomly selects study subjects at one point in time from a source population for cross-sectional survey and subsequent follow-up.

S5 Exhibit. Inapplicability of the complementary log model and the linear risk model for a case-control study of common diseases.

S1 Exhibit. Hypothesis testing for specified interaction classes in a complementary log regression.

Model (5) also permits hypothesis testing for specified interaction classes. A right-sided PRISM test, $\begin{cases} \mathrm{H}_0 : \beta_3 \le 0 \\ \mathrm{H}_1 : \beta_3 > 0 \end{cases}$, is a test for the presence of U_6 and U_9 [$\exp(\beta_3)$=PRISM >1 forbids $\mathrm{Risk}_{U_6} = \mathrm{Risk}_{U_9} = 0$], and a left-sided one, $\begin{cases} \mathrm{H}_0 : \beta_3 \ge 0 \\ \mathrm{H}_1 : \beta_3 < 0 \end{cases}$, a test for the presence of U_7 and U_8 [$\exp(\beta_3)$=PRISM <1 forbids $\mathrm{Risk}_{U_7} = \mathrm{Risk}_{U_8} = 0$]. A specific test for U_6 is $\begin{cases} \mathrm{H}_0 : \beta_3 - \beta_0 \le 0 \\ \mathrm{H}_1 : \beta_3 - \beta_0 > 0 \end{cases}$, where $\exp(\beta_3 - \beta_0) = \frac{\exp(\beta_3)}{\exp(\beta_0)} = \frac{\mathrm{PRISM}}{\mathrm{Peril}_{0,0}} = \frac{\mathrm{Peril}_{U_6}}{\mathrm{Peril}_{U_1} \times \mathrm{Peril}_{U_3} \times \mathrm{Peril}_{U_5} \times \mathrm{Peril}_{U_7} \times \mathrm{Peril}_{U_8}} > 1$ forbids $\mathrm{Risk}_{U_6} = 0$. A specific test for U_7 is $\begin{cases} \mathrm{H}_0 : -(\beta_0 + \beta_2 + \beta_3) \le 0 \\ \mathrm{H}_1 : -(\beta_0 + \beta_2 + \beta_3) > 0 \end{cases}$, where

$$\exp\left[-(\beta_0 + \beta_2 + \beta_3)\right] = \frac{1}{\mathrm{PRISM} \times \mathrm{Peril}_{0,1}} = \frac{\mathrm{Peril}_{U_7}}{\mathrm{Peril}_{U_1} \times \mathrm{Peril}_{U_3} \times \mathrm{Peril}_{U_4} \times \mathrm{Peril}_{U_6} \times \mathrm{Peril}_{U_9}} > 1 \text{ forbids}$$

$\mathrm{Risk}_{U_7} = 0$. A specific test for U_8 is $\begin{cases} \mathrm{H}_0 : -(\beta_0 + \beta_1 + \beta_3) \le 0 \\ \mathrm{H}_1 : -(\beta_0 + \beta_1 + \beta_3) > 0 \end{cases}$, where

$$\exp\left[-(\beta_0 + \beta_1 + \beta_3)\right] = \frac{1}{\mathrm{PRISM} \times \mathrm{Peril}_{1,0}} = \frac{\mathrm{Peril}_{U_8}}{\mathrm{Peril}_{U_1} \times \mathrm{Peril}_{U_2} \times \mathrm{Peril}_{U_5} \times \mathrm{Peril}_{U_6} \times \mathrm{Peril}_{U_9}} > 1 \text{ forbids}$$

$\mathrm{Risk}_{U_8} = 0$. A specific test for U_9 is $\begin{cases} \mathrm{H}_0 : -(\beta_0 + \beta_1 + \beta_2) \le 0 \\ \mathrm{H}_1 : -(\beta_0 + \beta_1 + \beta_2) > 0 \end{cases}$, where

$$\exp\left[-(\beta_0 + \beta_1 + \beta_2)\right] = \frac{\mathrm{Peril}_{0,0}}{\mathrm{Peril}_{1,0} \times \mathrm{Peril}_{0,1}} = \frac{\mathrm{Peril}_{U_9}}{\mathrm{Peril}_{U_1} \times \mathrm{Peril}_{U_2} \times \mathrm{Peril}_{U_4} \times \mathrm{Peril}_{U_7} \times \mathrm{Peril}_{U_8}} > 1 \text{ forbids}$$

$\mathrm{Risk}_{U_9} = 0$.

S2 Exhibit. Conducting the PRISM test based on the logistic, the probit, and the complementary log-log regressions.

For people in the population with an exposure profile of $X=x$ and $Z=z$ for each and every $x,z\in\{0,1\}$, let $\pi_{x,z}$ denote the cumulative disease risk (probability) in $(0,\ T)$. Assume that the disease risks follow a generalized linear model:

$$g\left(\pi_{x,z}\right)=\beta_0+\beta_1 x+\beta_2 z+\beta_3 xz,$$

where $g(\cdot)$ is a link function. To conduct a PRISM test, one first calculates the logarithm of the PRISM index,

$$\log \text{PRISM} = \log \text{Peril}_{1,1} - \log \text{Peril}_{1,0} - \log \text{Peril}_{0,1} + \log \text{Peril}_{0,0},$$

and its variance (in log scale),

$$\begin{aligned}\text{Var}\left(\log \text{PRISM}\right) &= \text{Var}\left(\log \text{Peril}_{1,1} - \log \text{Peril}_{1,0} - \log \text{Peril}_{0,1} + \log \text{Peril}_{0,0}\right)\\ &= \text{Var}\left(\log \text{Peril}_{1,1}\right)+\text{Var}\left(\log \text{Peril}_{1,0}\right)+\text{Var}\left(\log \text{Peril}_{0,1}\right)+\text{Var}\left(\log \text{Peril}_{0,0}\right).\end{aligned}$$

(There is no covariance term in the above variance formula, because the perils for the four different exposure profiles are independent of one another.) Then, one performs the following test:

$$Z=\frac{\log \text{PRISM}}{\sqrt{\text{Var}(\log \text{PRISM})}}\sim N\left(0,1\right).$$

The model-based estimates of $\log \text{Peril}$'s and $\text{Var}\left(\log \text{Peril}\right)$'s are detailed below for the logistic, the probit, and the complementary log-log regressions, respectively. The variances of simple sums of the beta coefficients are straightforward: $\text{Var}\left(\beta_0+\beta_1\right)=\mathbf{L}_{1,0}\mathbf{\Sigma}\mathbf{L}_{1,0}^{\mathbf{t}}$ with $\mathbf{L}_{1,0}=\left[1\ \ 1\ \ 0\ \ 0\right]$, $\text{Var}\left(\beta_0+\beta_2\right)=\mathbf{L}_{0,1}\mathbf{\Sigma}\mathbf{L}_{0,1}^{\mathbf{t}}$ with $\mathbf{L}_{0,1}=\left[1\ \ 0\ \ 1\ \ 0\right]$,

$\mathrm{Var}(\beta_0+\beta_1+\beta_2+\beta_3)=\mathbf{L}_{1,1}\mathbf{\Sigma}\mathbf{L}_{1,1}{}^{\mathbf{t}}$ with $\mathbf{L}_{1,1}=[1\ \ 1\ \ 1\ \ 1]$, respectively, where $\mathbf{\Sigma}$ is the variance-covariance matrix of the beta coefficients as estimated from the respective models. Most statistical software can output the variance of any user-specified linear combination of the beta coefficients, and one does not really have to perform the above matrix computations by hand. However, $\log \mathrm{Peril}$'s are non-linear functions of the beta coefficients. Here, the delta method is used to approximate their variances. (To our knowledge, there is no statistical software that can automatically output the variance of a user-specified non-linear function of the beta coefficients.)

(I) Logistic Regression:

$$g(\pi_{x,z})=\log\left(\frac{\pi_{x,z}}{1-\pi_{x,z}}\right)=\beta_0+\beta_1 x+\beta_2 z+\beta_3 xz,$$

$$\log \mathrm{Peril}_{1,1}=\log(1-\pi_{1,1})^{-1}=\log\left(1+e^{\beta_0+\beta_1+\beta_2+\beta_3}\right),$$

$$\log \mathrm{Peril}_{1,0}=\log(1-\pi_{1,0})^{-1}=\log\left(1+e^{\beta_0+\beta_1}\right),$$

$$\log \mathrm{Peril}_{0,1}=\log(1-\pi_{0,1})^{-1}=\log\left(1+e^{\beta_0+\beta_2}\right),$$

$$\log \mathrm{Peril}_{0,0}=\log(1-\pi_{0,0})^{-1}=\log\left(1+e^{\beta_0}\right),$$

$$\begin{aligned}\mathrm{Var}(\log \mathrm{Peril}_{1,1})=\mathrm{Var}\left[\log\left(1+e^{\beta_0+\beta_1+\beta_2+\beta_3}\right)\right]&\approx\left[\frac{d\log\left(1+e^{\beta_0+\beta_1+\beta_2+\beta_3}\right)}{d(\beta_0+\beta_1+\beta_2+\beta_3)}\right]^2\times\mathrm{Var}(\beta_0+\beta_1+\beta_2+\beta_3)\\&=\left(\frac{e^{\beta_0+\beta_1+\beta_2+\beta_3}}{1+e^{\beta_0+\beta_1+\beta_2+\beta_3}}\right)^2\times\mathrm{Var}(\beta_0+\beta_1+\beta_2+\beta_3),\end{aligned}$$

$$\operatorname{Var}\left(\log \text{Peril}_{1,0}\right)=\operatorname{Var}\left[\log\left(1+e^{\beta_0+\beta_1}\right)\right]\approx\left[\frac{d\log\left(1+e^{\beta_0+\beta_1}\right)}{d\left(\beta_0+\beta_1\right)}\right]^2\times\operatorname{Var}\left(\beta_0+\beta_1\right)$$

$$=\left(\frac{e^{\beta_0+\beta_1}}{1+e^{\beta_0+\beta_1}}\right)^2\times\operatorname{Var}\left(\beta_0+\beta_1\right),$$

$$\operatorname{Var}\left(\log \text{Peril}_{0,1}\right)=\operatorname{Var}\left[\log\left(1+e^{\beta_0+\beta_2}\right)\right]\approx\left[\frac{d\log\left(1+e^{\beta_0+\beta_2}\right)}{d\left(\beta_0+\beta_1\right)}\right]^2\times\operatorname{Var}\left(\beta_0+\beta_2\right)$$

$$=\left(\frac{e^{\beta_0+\beta_2}}{1+e^{\beta_0+\beta_2}}\right)^2\times\operatorname{Var}\left(\beta_0+\beta_2\right),$$

$$\operatorname{Var}\left(\log \text{Peril}_{0,0}\right)=\operatorname{Var}\left[\log\left(1+e^{\beta_0}\right)\right]\approx\left[\frac{d\log\left(1+e^{\beta_0}\right)}{d\beta_0}\right]^2\times\operatorname{Var}\left(\beta_0\right)$$

$$=\left(\frac{e^{\beta_0}}{1+e^{\beta_0}}\right)^2\times\operatorname{Var}\left(\beta_0\right).$$

(II) Probit Regression:

Let Φ be the cumulative distribution function of the standard normal distribution, and $\varphi=\Phi'$ be the probability density function of the standard normal distribution.

$$g\left(\pi_{x,z}\right)=\Phi^{-1}\left(\pi_{x,z}\right)=\beta_0+\beta_1 x+\beta_2 z+\beta_3 xz,$$

$$\log \text{Peril}_{1,1}=\log\left(1-\pi_{1,1}\right)^{-1}=-\log\left[1-\Phi\left(\beta_0+\beta_1+\beta_2+\beta_3\right)\right],$$

$$\log \text{Peril}_{1,0}=\log\left(1-\pi_{1,0}\right)^{-1}=-\log\left[1-\Phi\left(\beta_0+\beta_1\right)\right],$$

$$\log \text{Peril}_{0,1}=\log\left(1-\pi_{0,1}\right)^{-1}=-\log\left[1-\Phi\left(\beta_0+\beta_2\right)\right],$$

$$\log \text{Peril}_{0,0}=\log\left(1-\pi_{0,0}\right)^{-1}=-\log\left[1-\Phi\left(\beta_0\right)\right],$$

$$\begin{aligned}\operatorname{Var}\left(\log \text{Peril}_{1,1}\right) &= \operatorname{Var}\left(-\log\left[1-\Phi\left(\beta_0+\beta_1+\beta_2+\beta_3\right)\right]\right) \\ &\approx \left[-\frac{d\log\left[1-\Phi\left(\beta_0+\beta_1+\beta_2+\beta_3\right)\right]}{d\left(\beta_0+\beta_1+\beta_2+\beta_3\right)}\right]^2 \times \operatorname{Var}\left(\beta_0+\beta_1+\beta_2+\beta_3\right) \\ &= \left[\frac{\varphi\left(\beta_0+\beta_1+\beta_2+\beta_3\right)}{1-\Phi\left(\beta_0+\beta_1+\beta_2+\beta_3\right)}\right]^2 \times \operatorname{Var}\left(\beta_0+\beta_1+\beta_2+\beta_3\right),\end{aligned}$$

$$\begin{aligned}\operatorname{Var}\left(\log \text{Peril}_{1,0}\right) &= \operatorname{Var}\left(-\log\left[1-\Phi\left(\beta_0+\beta_1\right)\right]\right) \\ &\approx \left[-\frac{d\log\left[1-\Phi\left(\beta_0+\beta_1\right)\right]}{d\left(\beta_0+\beta_1\right)}\right]^2 \times \operatorname{Var}\left(\beta_0+\beta_1\right) \\ &= \left[\frac{\varphi\left(\beta_0+\beta_1\right)}{1-\Phi\left(\beta_0+\beta_1\right)}\right]^2 \times \operatorname{Var}\left(\beta_0+\beta_1\right),\end{aligned}$$

$$\begin{aligned}\operatorname{Var}\left(\log \text{Peril}_{0,1}\right) &= \operatorname{Var}\left(-\log\left[1-\Phi\left(\beta_0+\beta_2\right)\right]\right) \\ &\approx \left[-\frac{d\log\left[1-\Phi\left(\beta_0+\beta_2\right)\right]}{d\left(\beta_0+\beta_2\right)}\right]^2 \times \operatorname{Var}\left(\beta_0+\beta_2\right) \\ &= \left[\frac{\varphi\left(\beta_0+\beta_2\right)}{1-\Phi\left(\beta_0+\beta_2\right)}\right]^2 \times \operatorname{Var}\left(\beta_0+\beta_2\right),\end{aligned}$$

$$\begin{aligned}\operatorname{Var}\left(\log \text{Peril}_{0,0}\right) &= \operatorname{Var}\left(-\log\left[1-\Phi\left(\beta_0\right)\right]\right) \\ &\approx \left[-\frac{d\log\left[1-\Phi\left(\beta_0\right)\right]}{d\beta_0}\right]^2 \times \operatorname{Var}\left(\beta_0\right) \\ &= \left[\frac{\varphi\left(\beta_0\right)}{1-\Phi\left(\beta_0\right)}\right]^2 \times \operatorname{Var}\left(\beta_0\right).\end{aligned}$$

(III) Complementary Log-Log Regression:

$$g\left(\pi_{x,z}\right) = \log\left(-\log\left(1-\pi_{x,z}\right)\right) = \beta_0+\beta_1 x+\beta_2 z+\beta_3 xz,$$

$$\log \text{Peril}_{1,1} = \log\left(1-\pi_{1,1}\right)^{-1} = \log e^{e^{\beta_0+\beta_1+\beta_2+\beta_3}} = e^{\beta_0+\beta_1+\beta_2+\beta_3},$$

$$\log \text{Peril}_{1,0} = \log\left(1-\pi_{1,0}\right)^{-1} = \log e^{e^{\beta_0+\beta_1}} = e^{\beta_0+\beta_1},$$

$$\log \text{Peril}_{0,1} = \log\left(1-\pi_{0,1}\right)^{-1} = \log e^{e^{\beta_0+\beta_2}} = e^{\beta_0+\beta_2},$$

$$\log \text{Peril}_{0,0} = \log\left(1-\pi_{0,0}\right)^{-1} = \log e^{e^{\beta_0}} = e^{\beta_0}$$

$$\begin{aligned}\text{Var}\left(\log \text{Peril}_{1,1}\right) = \text{Var}\left(e^{\beta_0+\beta_1+\beta_2+\beta_3}\right) &\approx \left[\frac{d\left(e^{\beta_0+\beta_1+\beta_2+\beta_3}\right)}{d\left(\beta_0+\beta_1+\beta_2+\beta_3\right)}\right]^2 \times \text{Var}\left(\beta_0+\beta_1+\beta_2+\beta_3\right) \\ &= \left(e^{\beta_0+\beta_1+\beta_2+\beta_3}\right)^2 \times \text{Var}\left(\beta_0+\beta_1+\beta_2+\beta_3\right),\end{aligned}$$

$$\begin{aligned}\text{Var}\left(\log \text{Peril}_{1,0}\right) = \text{Var}\left(e^{\beta_0+\beta_1}\right) &\approx \left[\frac{d\left(e^{\beta_0+\beta_1}\right)}{d\left(\beta_0+\beta_1\right)}\right]^2 \times \text{Var}\left(\beta_0+\beta_1\right) \\ &= \left(e^{\beta_0+\beta_1}\right)^2 \times \text{Var}\left(\beta_0+\beta_1\right),\end{aligned}$$

$$\begin{aligned}\text{Var}\left(\log \text{Peril}_{0,1}\right) = \text{Var}\left(e^{\beta_0+\beta_2}\right) &\approx \left[\frac{d\left(e^{\beta_0+\beta_2}\right)}{d\left(\beta_0+\beta_2\right)}\right]^2 \times \text{Var}\left(\beta_0+\beta_2\right) \\ &= \left(e^{\beta_0+\beta_2}\right)^2 \times \text{Var}\left(\beta_0+\beta_2\right),\end{aligned}$$

$$\begin{aligned}\text{Var}\left(\log \text{Peril}_{0,0}\right) = \text{Var}\left(e^{\beta_0}\right) &\approx \left[\frac{d\left(e^{\beta_0}\right)}{d\beta_0}\right]^2 \times \text{Var}\left(\beta_0\right) \\ &= \left(e^{\beta_0}\right)^2 \times \text{Var}\left(\beta_0\right).\end{aligned}$$

S3 Exhibit. R code for complementary log regression.

```
##########################################################

###    To run a complementary log regression,                    ###

###    first construct a user-defined function, clog(), as below,      ###

###    and then make a statement, family=binomial(clog()),         ###

###    in the built-in function, glm().                            ###

#########################################################

clog <- function()
{     linkfun <- function(mu) -log(1-mu)
      linkinv <- function(eta) 1-exp(-eta)
      mu.eta <- function(eta) exp(-eta)
      valideta <- function(eta) TRUE
      link <- "linkinv"
      structure(list(linkfun = linkfun, linkinv = linkinv,mu.eta=mu.eta,
                        valideta = valideta, name = link),class = "link-glm")
}

glm(formula, family=binomial(clog()),….)
```

S4 Exhibit. Applicability of the complementary log regression for a sub-cohort study which randomly selects study subjects at one point in time from a source population for cross-sectional survey and subsequent follow-up.

Let $\mathbf{x}$ be a row vector as the exposure profile of a subject, and D be the disease status of a subject (coded as 1 for diseased and 0 for non-diseased). Assume that the disease risks in the source population follow a complementary log regression model as detailed below,

$$-\log\left[1-\Pr\left(D=1|\mathbf{x}\right)\right]=-\log\left[\Pr\left(D=0|\mathbf{x}\right)\right]=\alpha+\mathbf{x\beta},$$

where α is the baseline log peril and $\boldsymbol{\beta}$ is a column vector of parameters of interest. Let $R=1$ indicate the event that a person is recruited for study from the source population. Assume that the recruitment probability for each and every individual in the population is the same. We thus have that,

$$\begin{aligned}-\log\left[1-\Pr\left(D=1|\mathbf{x},R=1\right)\right]&=-\log\left[\Pr\left(D=0|\mathbf{x},R=1\right)\right]\\&=-\log\left[\frac{\Pr\left(R=1|D=0,\mathbf{x}\right)\times\Pr\left(D=0|\mathbf{x}\right)\times\Pr\left(\mathbf{x}\right)}{\Pr\left(R=1|\mathbf{x}\right)\times\Pr\left(\mathbf{x}\right)}\right]\\&=-\log\left[\frac{\Pr\left(R=1|D=0,\mathbf{x}\right)}{\Pr\left(R=1|\mathbf{x}\right)}\right]-\log\left[\Pr\left(D=0|\mathbf{x}\right)\right]\\&=0-\log\left[1-\Pr\left(D=1|\mathbf{x}\right)\right]\\&=\alpha+\mathbf{x\beta},\end{aligned}$$

which is the same complementary log regression model as in the source population.

S5 Exhibit. Inapplicability of the complementary log model and the linear risk model for a case-control study of common diseases.

Let $\mathbf{x}$ be a row vector as the exposure profile of a subject, and D be the disease status of a subject (coded as 1 for diseased and 0 for non-diseased). Let $R=1$ indicate the event that a person is recruited for a case-control study from the source population, with the following sampling schemes, $\Pr\left(R=1 \middle| D=1,\mathbf{x}\right)=\phi_1$ and $\Pr\left(R=1 \middle| D=0,\mathbf{x}\right)=\phi_0$, for 'case' and 'control', respectively.

Assume first that the disease risks in the source population follow a complementary log regression model,

$$-\log\left[1-\Pr\left(D=1 \middle| \mathbf{x}\right)\right]=-\log\left[\Pr\left(D=0 \middle| \mathbf{x}\right)\right]=\alpha+\mathbf{x}\boldsymbol{\beta},$$

where α is the baseline log peril (considered as a nuisance parameter here for a case-control study) and $\boldsymbol{\beta}$ is a column vector of parameters of interest. In the case-control data, we have that,

$$
\begin{aligned}
-\log\left[1-\Pr\left(D=1|\mathbf{x},R=1\right)\right] &= \log\left[\Pr^{-1}\left(D=0|\mathbf{x},R=1\right)\right] \\
&= \log\left[\frac{\Pr\left(R=1|\mathbf{x}\right)\times\Pr\left(\mathbf{x}\right)}{\Pr\left(R=1|D=0,\mathbf{x}\right)\times\Pr\left(D=0|\mathbf{x}\right)\times\Pr\left(\mathbf{x}\right)}\right] \\
&= \log\left[\frac{\Pr\left(R=1,D=1|\mathbf{x}\right)+\Pr\left(R=1,D=0|\mathbf{x}\right)}{\Pr\left(R=1|D=0,\mathbf{x}\right)\times\Pr\left(D=0|\mathbf{x}\right)}\right] \\
&= \log\left[\frac{\begin{array}{c}\Pr\left(R=1|D=1,\mathbf{x}\right)\times\Pr\left(D=1|\mathbf{x}\right)\\ +\Pr\left(R=1|D=0,\mathbf{x}\right)\times\Pr\left(D=0|\mathbf{x}\right)\end{array}}{\Pr\left(R=1|D=0,\mathbf{x}\right)\times\Pr\left(D=0|\mathbf{x}\right)}\right] \\
&= \log\left[\frac{\phi_1\times\left(1-\Pr\left(D=0|\mathbf{x}\right)\right)+\phi_0\times\Pr\left(D=0|\mathbf{x}\right)}{\phi_0\times\Pr\left(D=0|\mathbf{x}\right)}\right] \\
&= \log\left[\frac{\phi_0-\phi_1}{\phi_0}+\frac{\phi_1}{\phi_0}\Pr^{-1}\left(D=0|\mathbf{x}\right)\right] \\
&= \log\left[\frac{\phi_0-\phi_1}{\phi_0}+\frac{\phi_1}{\phi_0}\times\exp\left(\alpha+\mathbf{x}\boldsymbol{\beta}\right)\right].
\end{aligned}
$$

This is not a complementary log regression model unless $\phi_1=\phi_0$.

Next, assume that the disease risks in the source population follow a linear risk model,

$$\Pr\left(D=1|\mathbf{x}\right)=\alpha+\mathbf{x}\boldsymbol{\beta},$$

where α is the baseline risk (considered again as a nuisance parameter for a case-control study) and $\boldsymbol{\beta}$ is a column vector of parameters of interest. In the case-control data, we have that,

$$
\begin{aligned}
\Pr\left(D=1\middle|\mathbf{x},R=1\right) &= \frac{\Pr\left(R=1\middle|D=1,\mathbf{x}\right)\times\Pr\left(D=1\middle|\mathbf{x}\right)\times\Pr\left(\mathbf{x}\right)}{\Pr\left(R=1\middle|\mathbf{x}\right)\times\Pr\left(\mathbf{x}\right)} \\
&= \frac{\Pr\left(R=1\middle|D=1,\mathbf{x}\right)\times\Pr\left(D=1\middle|\mathbf{x}\right)}{\Pr\left(R=1,D=1\middle|\mathbf{x}\right)+\Pr\left(R=1,D=0\middle|\mathbf{x}\right)} \\
&= \frac{\Pr\left(R=1\middle|D=1,\mathbf{x}\right)\times\Pr\left(D=1\middle|\mathbf{x}\right)}{\Pr\left(R=1\middle|D=1,\mathbf{x}\right)\times\Pr\left(D=1\middle|\mathbf{x}\right)+\Pr\left(R=1\middle|D=0,\mathbf{x}\right)\times\Pr\left(D=0\middle|\mathbf{x}\right)} \\
&= \frac{\phi_1\times\Pr\left(D=1\middle|\mathbf{x}\right)}{\phi_1\times\Pr\left(D=1\middle|\mathbf{x}\right)+\phi_0\times\left(1-\Pr\left(D=1\middle|\mathbf{x}\right)\right)} \\
&= \frac{\phi_1\times\Pr\left(D=1\middle|\mathbf{x}\right)}{\phi_0+\left(\phi_1-\phi_0\right)\times\Pr\left(D=1\middle|\mathbf{x}\right)} \\
&= \frac{\phi_1\times\left(\alpha+\mathbf{x}\boldsymbol{\beta}\right)}{\phi_0+\left(\phi_1-\phi_0\right)\times\left(\alpha+\mathbf{x}\boldsymbol{\beta}\right)} \\
&= \frac{\alpha+\mathbf{x}\boldsymbol{\beta}}{\left[\phi_0/\phi_1+\left(1-\phi_0/\phi_1\right)\times\alpha\right]+\left(1-\phi_0/\phi_1\right)\times\mathbf{x}\boldsymbol{\beta}}.
\end{aligned}
$$

Again, this is not a linear risk model unless $\phi_1=\phi_0$.
